# Salivary PYY: A Putative Bypass to Satiety

**DOI:** 10.1371/journal.pone.0026137

**Published:** 2011-10-10

**Authors:** Andres Acosta, Maria D. Hurtado, Oleg Gorbatyuk, Michael La Sala, David Duncan, George Aslanidi, Martha Campbell-Thompson, Lei Zhang, Herbert Herzog, Antonis Voutetakis, Bruce J. Baum, Sergei Zolotukhin

**Affiliations:** 1 Pediatrics, University of Florida, Gainesville, Florida, United States of America; 2 Pathology, University of Florida, Gainesville, Florida, United States of America; 3 Molecular Genetics and Microbiology, University of Florida, Gainesville, Florida, United States of America; 4 Garvan Institute of Medical Research, Sydney, Australia; 5 Molecular Physiology and Therapeutics Branch, National Institute of Dental and Craniofacial Research (NIDCR), National Institutes of Health (NIH), Bethesda, Maryland, United States of America; National Taiwan University Hospital, Taiwan

## Abstract

Peptide YY_3-36_ is a satiation hormone released postprandially into the bloodstream from L-endocrine cells in the gut epithelia. In the current report, we demonstrate PYY_3-36_ is also present in murine as well as in human saliva. In mice, salivary PYY_3-36_ derives from plasma and is also synthesized in the taste cells in taste buds of the tongue. Moreover, the cognate receptor Y2R is abundantly expressed in the basal layer of the progenitor cells of the tongue epithelia and von Ebner's gland. The acute augmentation of salivary PYY_3-36_ induced stronger satiation as demonstrated in feeding behavioral studies. The effect is mediated through the activation of the specific Y2 receptor expressed in the lingual epithelial cells. In a long-term study involving diet-induced obese (DIO) mice, a sustained increase in PYY_3-36_ was achieved using viral vector-mediated gene delivery targeting salivary glands. The chronic increase in salivary PYY_3-36_ resulted in a significant long-term reduction in food intake (FI) and body weight (BW). Thus this study provides evidence for new functions of the previously characterized gut peptide PYY_3-36_ suggesting a potential simple and efficient alternative therapeutic approach for the treatment of obesity.

## Introduction

A significant portion of metabolic polypeptides has been shown to be expressed in taste cells (TCs) or to be present in saliva. This long list now includes insulin, leptin, adiponectin glucagon, glucagon-like peptide-1 (GLP-1), cholecystokinin (CCK), neuropeptide Y (NPY), vasoactive intestinal peptide (VIP), ghrelin, and galanin [1], [2], [3], [4], [5], [6], [7], [8], [9], [10]. In addition, the cognate receptors for these peptide hormones are expressed in TCs or found in fibers of afferent taste nerves in oral mucosa [4], [6], [7], [9], [10], [11], [12], [13]. Anatomical proximity of agonists and receptors suggested their putative roles in taste functions. Indeed, most of these polypeptides have been implicated in modulation of different tastes such as sweet [4], [10], [11], [13], salty [14], sour [4], [14], and umami [15]. Little, however, is known whether these or other metabolic peptides that are present in saliva could play a more ‘traditional’ role regulating feeding behavior.

Peptide YY (PYY), a well-characterized molecular mediator of satiation, is released mostly by L-endocrine cells in the distal gut epithelia in response to the amount of calories ingested. The anorectic action of the truncated form PYY_3-36_ is apparently mediated through the inhibitory actions of its preferred Y2 receptor highly expressed in orexigenic NPY neurons in the hypothalamic arcuate nucleus (ARC). The acute peripheral administration of PYY_3-36_ resulted in significant reduction of food intake (FI) and body weight (BW) suggesting its potential therapeutic application for obesity treatment [16]. The latter results, however, could not be replicated by other groups [17] highlighting the necessity of a more detailed study of the functions of PYY_3-36_ in regulating feeding behavior and satiety.

The purpose of this investigation was to identify the presence and sources of a gut hormone PYY in saliva and to test whether salivary PYY could be utilized to modulate feeding behavior in mice.

## Results

### Murine and human saliva contains satiation hormone PYY

PYY is synthesized and secreted into the bloodstream mainly from entero-endocrine L cells in the distal gastrointestinal tract. Due to the rapid degradation, detection of this hormone in the serum poses a serious technical challenge, which can be improved by collecting samples in the presence of protease inhibitors. Using similar technique for samples of saliva collected from mice we have detected PYY in saliva at similar levels to those found in plasma (45+/−4.7 pg/ml and 81.4+/− 4.4 pg/ml, respectively).

**Figure 1:**
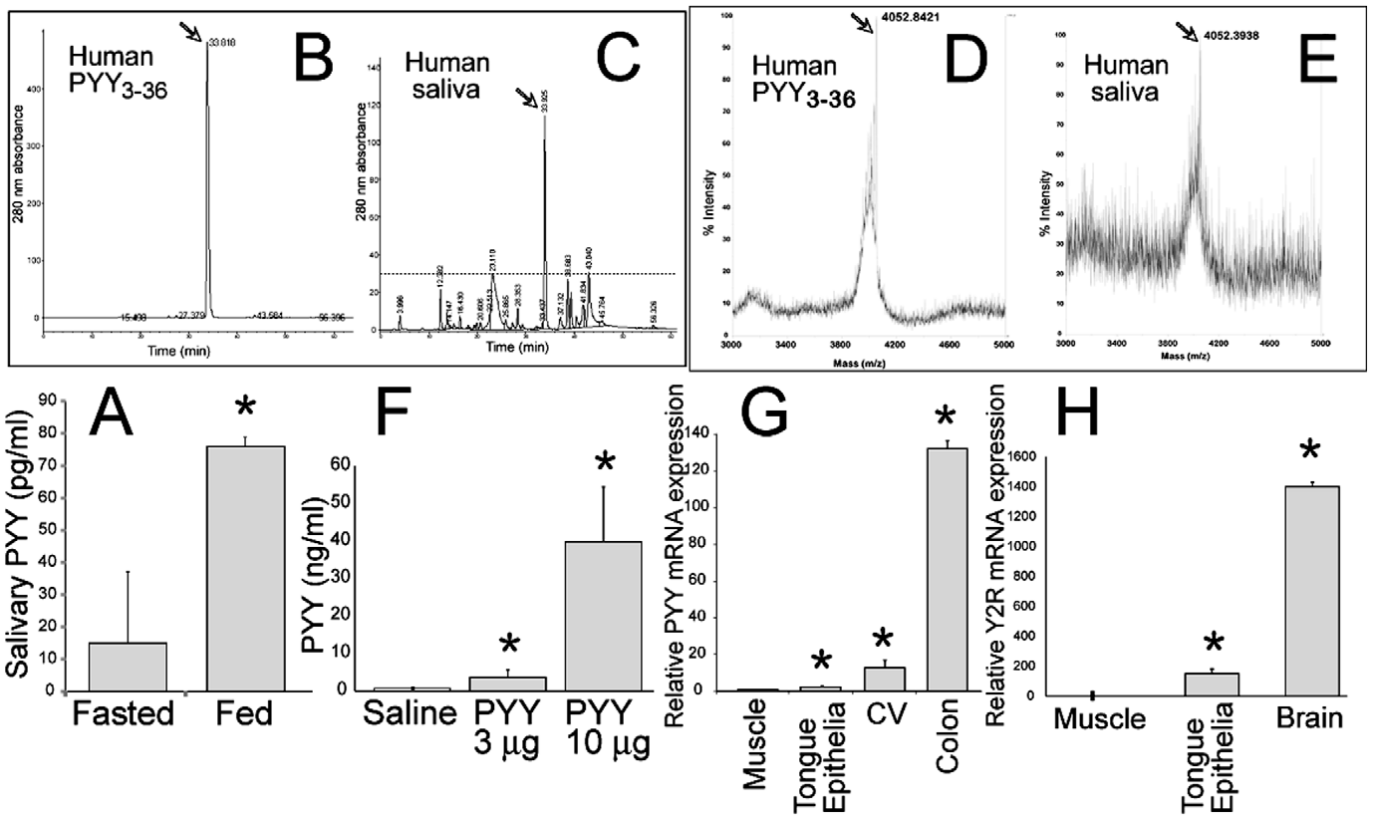
Characterization of PYY_3-36_ origins in human and of murine saliva. *(A)* Concentration of PYY_3-36_ in human saliva during fasting and 30 minutes after of finishing a standardized meal (n = 5), *P<0.05. *(B)* Separation of synthetic PYY_3-36_ by RP-HPLC. *(C)* Separation of human saliva by RP-HPLC. *(D)* Validation of synthetic PYY_3-36_ by MALDI-TOF *(E)* Validation of PYY_3-36_ from human saliva by MALDI-TOF. Arrows indicate PYY_3-36_ peaks. *(F)* PYY_3-36_ in saliva from male C57Bl/6J mice (n = 10 per group) injected with saline or synthetic murine PYY_3-36_ *i.p.*, at 10 min post injection. *(G)* RT-PCR assay measuring relative PYY mRNA expression in the muscle as a (-) control, tongue epithelium, and circumvallate papillae (CV) with colon as a (+) control in male C57Bl/6 mice (n = 10 per group). *(H)* RT-PCR assay measuring relative Y2R mRNA expression in the tongue epithelium, muscle as a (-) control, and brain as a (+) control in male C57Bl/6 mice (n = 8 per group). *P<0.05.

Likewise, PYY_3-36_ was detected in saliva samples of healthy human male volunteers that were fasted overnight (Fig. 1A). Interestingly, 30 min after consumption of a 450 kcal meal the concentration of PYY_3-36_ increased significantly suggesting a possible association between feeding and the concentration of PYY_3-36_ in saliva.

To validate the RIA results, we analyzed human PYY_3-36_ from saliva using mass spectrometry. To purify salivary PYY_3-36_, we utilized RP-HPLC using synthetic PYY_3-36_ as a positive control to calibrate the column (Fig. 1B). After RP-HPLC of a saliva sample, major peak (Fig. 1C) coinciding in its respective position in the elution profile of synthetic PYY_3-36_ was collected and further analyzed by MALDI-TOF. In both samples, synthetic (Fig. 1D) and salivary PYY_3-36_ (Fig. 1E), identical peaks of 4052 Da, a close experimental approximation of molecular weight for human PYY_3-36_ (4050 Da), were documented. We were not able to identify PYY_1-36_ in samples of human saliva using similar analysis (data not shown).

### Dual origin of salivary PYY

Some proteins enter saliva from salivary glands where they are expressed and secreted in an exocrine fashion via zymogen granules. Other peptides can enter saliva as a transudate from serum. To establish the source of salivary PYY_3-36_ we asked whether peptide circulating in a bloodstream can be transported into saliva. To this end, mice were injected intraperitoneally (*i.p.)* with increasing doses of murine synthetic PYY_3-36_ (Fig. 1F). Indeed, there was a significant relationship between the injected dose and the concentration of the peptide detected in saliva, substantiating human data (Fig. 1A) and indicating that a fraction of salivary PYY_3-36_ can be attributed to the circulating peptide.

**Figure 2:**
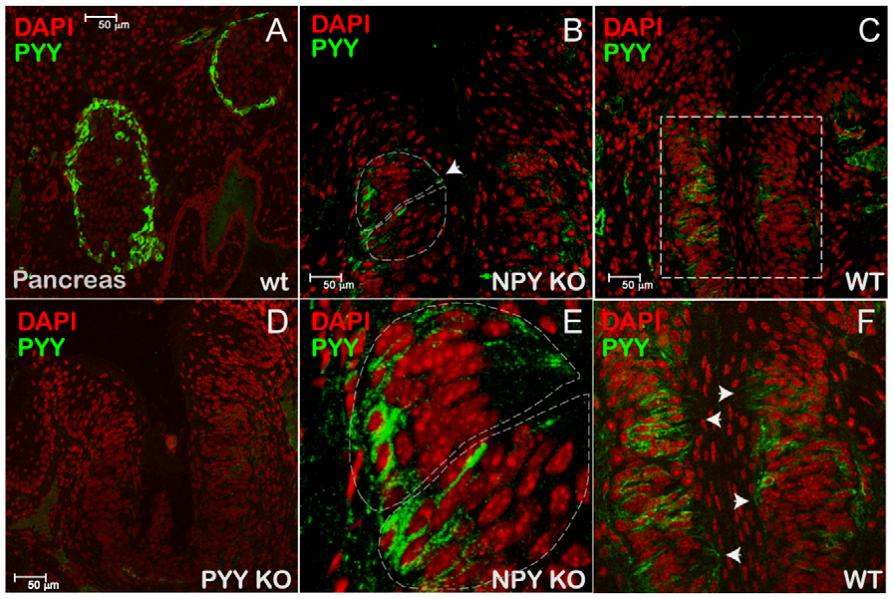
PYY is synthesized in taste cells. *(A)* Immunolocalization of PYY-positive cells in a-cells in the murine pancreas, a (+) control. *(B)* Immunolocalization of PYY in CV of a NPY KO mouse, a control for PYY antibodies cross-reactivity. *(C)* Immunolocalization of PYY in CV of a C57Bl/6J mouse (WT). *(D)* Immunolocalization of PYY in CV of a PYY KO mouse, a (-) control. *(E)* close-up of *(B)*. *(F)* close-up of *(C)*. Arrowheads point at the apical part of a taste bud.

To test whether PYY_3-36_ is also synthesized in the oral cavity, we have analyzed RNA isolated from murine tongue epithelia and from circumvallate papillae (CV) of the tongue. Both sources appear to contain PYY-specific messages with CV showing higher levels of expression (Fig. 1G). To validate PYY expression data we conducted immunolocalization analyses of CV. We used a-cells in pancreatic islands in C57Bl/6J mice as a positive control (Fig. 2A) and CV taste buds in PYY KO mice [18] as a negative control (Fig. 2D). We observed PYY-positive cells in the taste buds on both sides of the CV's sulcus (Fig. 2C, F). To exclude a potential cross-reactivity of PYY antibodies with NPY that had been previously shown to be expressed in TCs [7], we also used NPY KO mice [19] and detected strong PYY immunoreactivity in TCs in these mice as well (Fig. 2B, E). The PYY appears to be localized in secretion granules within TC cytoplasm (Fig. 2E) indicating its functional similarity to PYY secreted from the gut entero-endocrine L cells [20]. Collectively, these data suggest that salivary PYY_3-36_ originates from two independent sources: circulating plasma and cells in the taste buds.

### Y2R is expressed in the basal epithelial cells of the tongue

**Figure 3:**
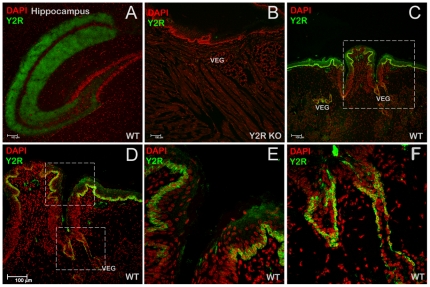
Y2 receptor is synthesized in the epithelial cells of the tongue. *(A)* Immunolocalization of Y2R-positive cells in the hippocampus of C57Bl/6J mouse (WT), a (+) control. *(B)* Immunolocalization of Y2R in the tongue epithelia of Y2R KO mouse, a (-) control. VEG – von Ebner's gland. *(C)* Immunolocalization of Y2R-positive cells in the CV area of the tongue of a C57Bl/6J mouse. *(D)* close-up of *(C)*. *(E)*, and *(F)* close ups of *(D)*, top and bottom rectangles, respectively.

To assess a possible functional role to salivary PYY, we have studied the expression profile of the PYY_3-36_-preferring receptor, Y2R. We detected significant levels of expression of Y2R mRNA by RT-PCR using mRNA isolated from murine tongue epithelia (Fig. 1H). The IHC analysis was conducted using brains from C57Bl/6J mice as a positive control [21] (Fig. 3A) and the tongue epithelia from Y2R KO as a negative control [22] (Fig. 3B). In wt C57Bl/6 mice, one layer of basal epithelia cells was strongly positive for Y2R (Fig. 3C, D, E). In addition, epithelial cells lining up ducts of the von Ebner's gland (VEG) expressed Y2R as well (Fig. 3C, D, F). We were unable to detect any Y2R in taste cells (data not shown).

**Figure 4:**
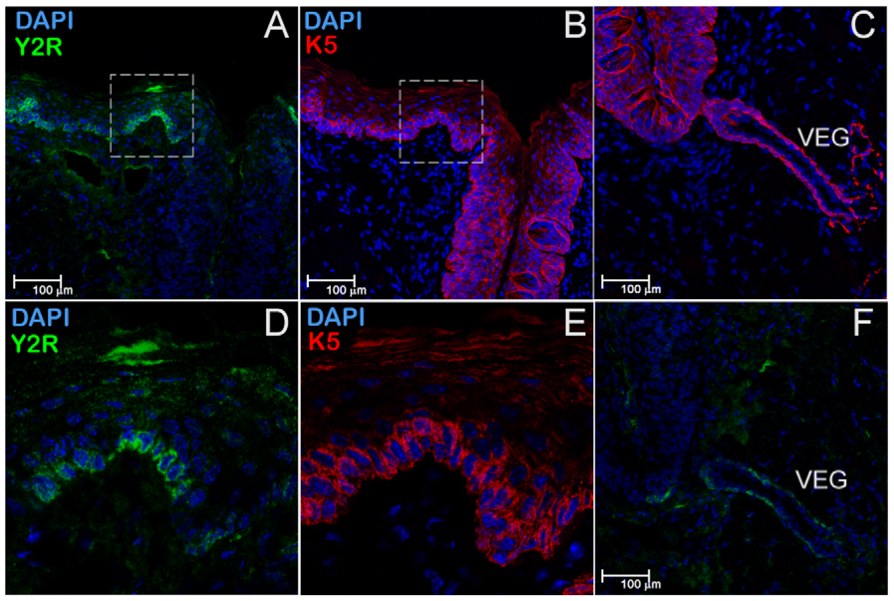
A subpopulation of epithelial progenitor cells in the tongue epithelia expresses Y2R. Two sequential mirror sections of the tongue were immunostained for Y2R *(A*, *D*, and and *F)*, or Cytokeratin 5 (K5) *(B*, *E*, and *C)*. For better viewing, K5 images were reflected horizontally. Areas at the sulcus edge, positive for both Y2R *(A)* and K5 *(B)* (dashed rectangles), are shown as close-up images in *(D)* and *(E)*, respectively. Panels *(C)* and *(F)* show Y2R and K5-positive cells in von Ebner's gland connecting to CV's sulcus.

**Figure 5:**
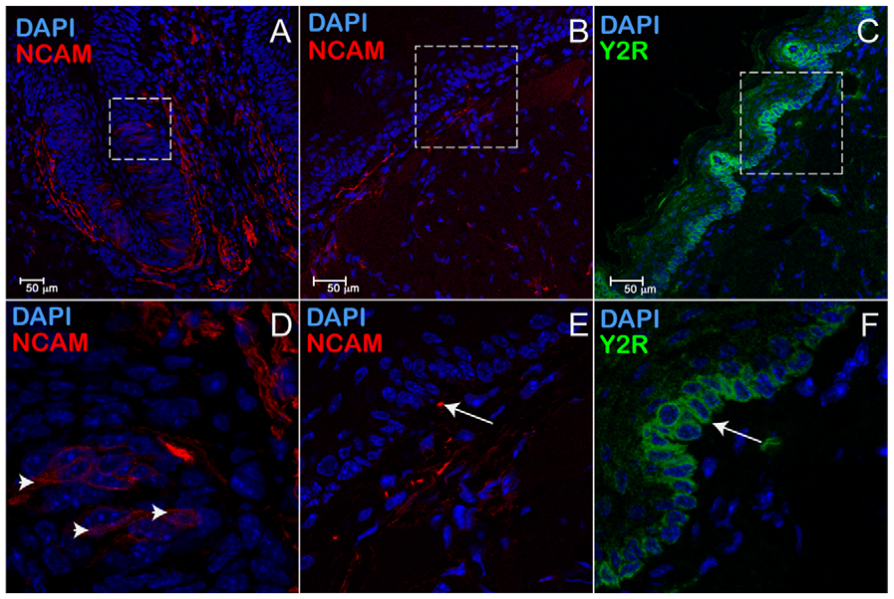
Neuronal filaments innervate circumvallate papillae (CV) as well as the basal layer of cells distant from CV. Immunostaining for NCAM in CV *(A)* shows subpopulation of taste cells expressing K5 (marked by arrowheads in panel D, a close-up from panel *A*), as well as a dense mesh of filaments at the basolateral surfaces of the taste buds. Rectangles in *(B)* and *(C)* designate the same areas in two sequential mirror sections stained for NCAM (red), or Y2R (green). The protrusions in the tongue epithelia surface *(B, C, E,* and *F)* are filiform papillae transversely sectioned. Even distant from CV, the Y2R-positive epithelial layer is morphologically close to neuro-filament layer below *(E)*. Some Y2R cells and NCAM filaments appear to be juxtaposed (arrows in *E* and *F*).

To establish the lineage identity of Y2R-positive cells we used cytokeratin-5 (K5), a basal cell epithelia marker in adult [23] and embryonic [24] salivary glands as well as a marker of progenitor cells of the filiform papillae [25]. Staining of sequential mirror sections with either Y2R or K5 antibodies (Fig. 4A, D, F, or Fig. 4B, C, E, respectively) revealed that Y2R is apparently expressed in a single apical layer of progenitor cells in the tongue epithelium (Fig. 4A, D) as well as in von Ebner's gland ducts and acini (Fig. 3C, D, F, and 4F), suggesting a possible trophic role of PYY signaling in mitotic signaling/regeneration. Coincidentally, the same apical layer of Y2R-positive cells appears to be innervated with neuron filaments as evident from the immunostaining using Neural Cell Adhesion Molecule (NCAM) neuronal marker antibodies (Fig. 5). The anatomical location of Y2R-positive cells, combined with their somatosensory innervation implied a possible functional role for salivary PYY_3-36_ ligand and its preferred Y2 receptor related to the regulation of feeding behavior.

### Oral PYY_3-36_ augmentation therapy

**Figure 6:**
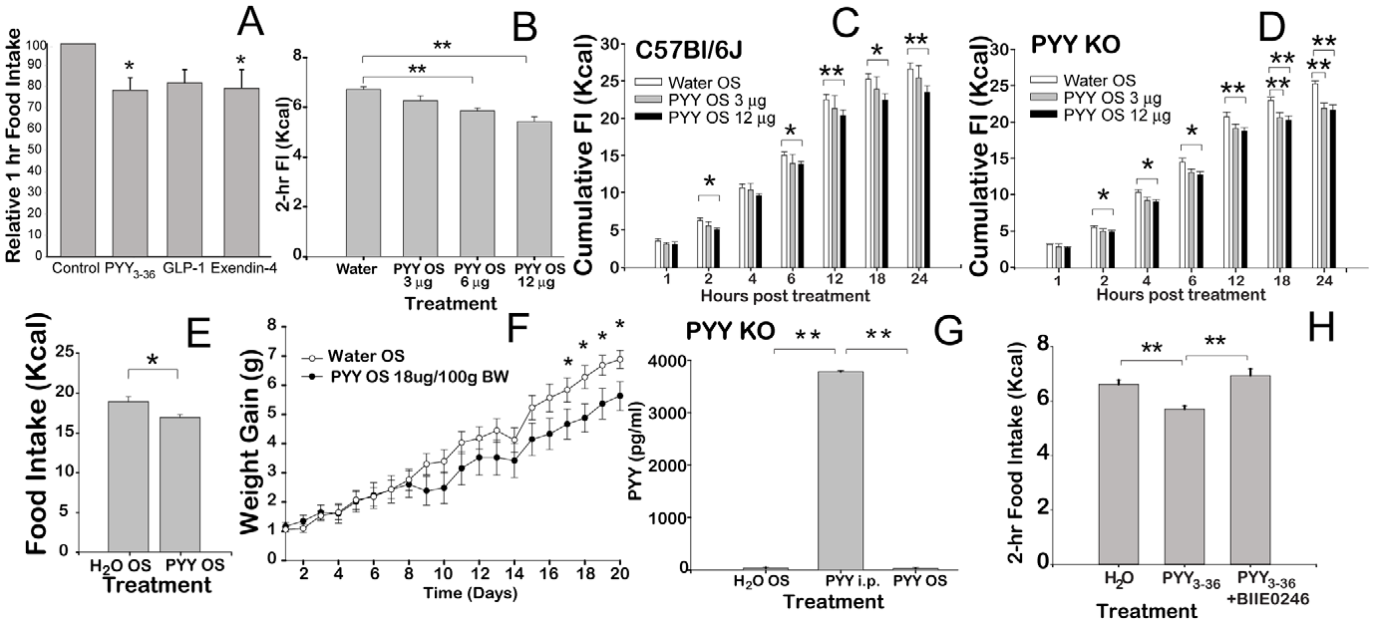
Oral PYY_3-36_ augmentation therapy. *(A)* Effect of PYY_3-36_ (3 mg/100 g BW), GLP-1(3 mg/100 g BW), or Exendin-4 (10 mg/100 g BW) oral spray (n = 10 each group) on 1 hour FI after 24 hours fasting compared to control oral spray (n = 10). Experiments for each peptide were conducted separately and the results were integrated in one graph as fractions of average amount of FI consumed by the control group. PYY_3-36_-, and Exendin-4-treated mice consumed significantly less food. *(B)* Dose-response effect of PYY_3-36_ on 2 hr FI vs. control (n = 8 each group). *(C)* Effect of PYY_3-36_ oral spray on FI in C57Bl/6J mice measured at 1, 2, 6, 12, 18, and 24 hr post treatment (n = 8/group). *(D)* Effect of PYY_3-36_ oral spray on FI in PYY^-/-^ mice measured at 1, 2, 6, 12, 18, and 24 hr post treatment (n = 8/group). *(E)* Average 24-hr FI in DIO mice treated with daily PYY_3-36_ OS (18 mg/100 g). *(F)* Effect of daily PYY_3-36_ OS (18 mg/100 g) treatment on BW change in DIO C57Bl/6J mice (n = 9 per each group). *(G)* Concentration of PYY_3-36_ in plasma of PYY KO mice 10 min after PYY_3-36_ (18 mg/100 g BW), or control oral spray vs. PYY_3-36_ injected *i.p.* (6 mg/100 g BW) (n = 10 per group). *(H)* Effect of Y2R specific antagonist BIIE0246 on anorexigenic action of PYY_3-36_ (n = 8 per each group) measured as 2 hr FI after 24 hr fast.*P<0.05, **P<0.01.

Intraperitoneal injection of PYY_3–36_ leading to higher circulating levels of the peptide results in a dose-dependent reduction in FI in rodents [16]. To test whether similar anorectic effect could be mediated through changes in salivary PYY_3-36_, we have developed a small spraying device that can be applied to the oral cavity in small rodent models. After 24 hours of fasting, the PYY_3-36_ treated group consumed, on average, 12.3% less food than the control group (Fig. 6A). In separate experiments, mice were also treated with GLP-1, or Exendin-4 peptides which were previously shown to induce anorectic responses. Similarly, these peptides administered by oral spray induced stronger satiation, which was also significant for Extendin-4. Additional dose-response studies showed that the increased doses of PYY_3-36_ led to a proportionate reduction in one hour FI, down to 81% of control values at the maximum applied dose of 12 mg/100 g (Fig. 6B). Using a similar approach, peptide amylin was tested by oral spray application resulting in no change of FI (data not shown).

To investigate the duration of action of orally applied PYY_3-36_ we conducted standard satiation studies in C57Bl/6J as well as in PYY KO mice measuring FI over 24 hours after single treatment. At the high treatment dose (12 mg/100 g BW) the anorexigenic response for both C57Bl/6J and PYY^-/-^ was significant until the next treatment 24 hrs later (Fig. 6C and 4D, respectively). However, when animals were treated with the low dose (3 mg/100 g BW), only PYY KO mice responded to the treatment in significant manner observed after 18 and 24 hrs post experiment (Fig. 6D).

Because of the sustained 24-hr effect, we tested whether repeated once-a-day treatment would also affect the FI and BW accumulation over extended period of time when animals were fed high fat (HF) diet *ad libitum*. Indeed, DIO mice treated with PYY_3-36_ oral spray (18 mg/100 g) consumed, on a daily basis, significantly less HF food (Fig. 6E) resulting in retarded BW accumulation which became significantly on day 17^th^ of the treatment (Fig. 6F).

The augmentation in oral PYY_3-36_ had a pronounced anorectic physiological effect similar to the previously described systemic augmentation. To verify that PYY_3-36_ applied by oral spray did not increase the circulating concentration of the peptide, we assayed the concentration of PYY_3-36_ in the plasma 10 min after oral spray (18 mg/100 g BW) treatment or after *i.p.* injection (6 mg/100g BW). To exclude potential interference with circulating background PYY in the plasma, we used PYY KO mice [18]. There was no detectable PYY_3-36_ in the plasma in the vehicle-, and PYY_3-36_-sprayed mice, while there was a significant increase in PYY_3-36_ found in plasma in the *i.p.*- injected animals implying that PYY_3-36_ applied by oral spray acted through its receptors expressed in the oral mucosa (Fig. 6G).

Direct proof of PYY_3-36_/Y2R interaction was obtained by utilizing a selective Y2 receptor antagonist BIIE0246 [26]. The antagonist and PYY_3-36_ agonist (12 mg/100 g BW) were mixed at the 50∶1 molar ratio, respectively, and the mixture was used to treat fasted mice as described above (Fig. 6H). Y2R antagonist completely ablated the anorexigenic effect of PYY_3-36_ (Fig 6H). The application of BIIE0246 alone had no effect on 2 hr FI (data not shown).

### Long-term increase in salivary PYY_3-36_ modulates FI and BW

**Figure 7:**
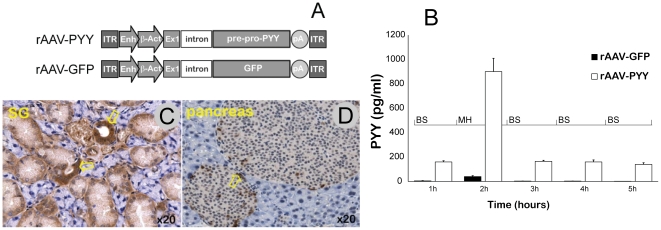
Validation of recombinant Adeno-associated virus encoding pre-pro-PYY (rAAV-PYY). *(A)* Diagram of rAAV-PYY and rAAV-GFP cassettes: ITR - inverted terminal repeats of rAAV serotype 2; CBA - Cytomegalovirus intermediate early enhancer sequence/chicken b-acting promoter; murine Pre-pro-Peptide YY cDNA, GFP - green fluorescence protein cDNA. *(B)* PYY secretion study in NCI-H716 cells. The experiment was performed on 3 different occasions with 10 wells per group. Media was assayed at the times indicated. BS – basal secretion; MH – secretion stimulated with 2% meat hydrolysate. *(C)* Cellular distribution of rAAV-PYY in salivary glands. Submandibular glands infused with rAAV-PYY removed 22 weeks after administration and evaluated for PYY expression by immunohistochemistry. The brown color indicates PYY-positive cells. Arrows indicate PYY-positive striated ducts. *(D)* Positive control – PYY-stained cells in murine pancreas.

Standard satiation studies in mice as conducted and described above, cannot be extrapolated to predict the changes in ingestive behavior in humans due to their very different feeding patterns. Because of their intense metabolism and high caloric requirements, feeding activities become a dominant part of murine behavior manifested in frequent meals when food provided *ad lib*. Consequently, employing a sporadic acute elevation in salivary PYY, such as obtained when using an oral spray, might not be an optimal way to modulate feeding behavior in mice. We, therefore, developed an alternative protocol to provide a sustained supply of exogenous PYY_3-36_ in saliva using gene delivery mediated by viral vector. The choice of targeted vector delivery was based on previous findings demonstrating efficient transduction of cells in the salivary gland (SG) using recombinant adeno-associated virus (rAAV) [27]. It was anticipated that regulated secretory pathway proteins such as PYY, when delivered as transgenes to SG, would be secreted predominantly into saliva [28]. To this end, we constructed rAAV encoding pre-pro-peptide YY (rAAV-PYY) (Fig. 7A).

To verify whether the transgene encoding a precursor generates a functionally adequate PYY, we utilized the human NCI-H716 intestinal cell line [29] cultured *in vitro*. Cells were transduced with rAAV-PYY and then were differentiated to display endocrine features, in particular the formation of granule-like compartments. In the basal state, cells transduced with rAAV-PYY secreted twice as much PYY, but when induced with 2% meat hydrolysate (MH), the secretion level increased by 100-fold suggesting that transgene-encoded PYY accumulates and undergoes secretion in physiologically regulated fashion (Fig. 7B).

**Figure 8:**
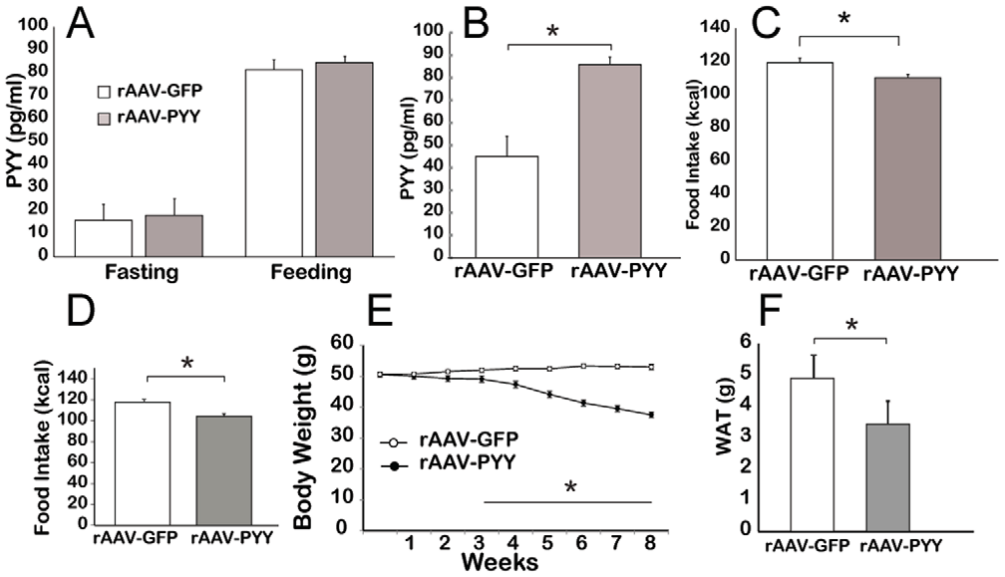
Effect of PYY gene transfer to the salivary glands in C57Bl/6J mice. *(A)* Concentration of PYY_3-36_ in plasma during fasting and after feeding. *(B)* Concentration of PYY_3-36_ in saliva. *(C)* Effect of PYY_3-36_ salivary gland gene delivery on weekly FI in mice fed HF diet. *(D)* Effect of PYY_3-36_ salivary gland gene delivery on weekly FI in DIO mice. *(E)* BW change in DIO C57Bl/6J treated with rAAV-GFP (control group) and rAAV-PYY over 8 weeks post injection. *(F)* Weight of visceral white adipose tissue at 8 weeks post injection in mice treated with rAAV (all groups were 10 animals/group) *P<0.05.

Next we tested the efficiency of PYY gene transfer *in vivo* C57Bl/5J mice. One month after vector administration, levels of PYY had been assayed in the blood and in saliva. While there was no significant difference in plasma PYY levels during fasting and after feeding after SG gene transfer (Fig. 8A), there was a 2-fold increase in salivary PYY levels (Fig. 8B). Immunohistochemistry analysis validated the transduction of cells in the salivary gland showing PYY-positive cells in ductal cells (Fig. 7C), the gland cell type previously shown transduced by rAAV2 vectors [27]. Apparently, this modest increase in salivary PYY was sufficient to significantly reduce weekly average calorie consumption (Fig. 8C).

Next, we asked whether a long-term increase in salivary PYY would reduce FI and, perhaps, BW in mice rendered obese by a high fat diet (HF, 60% calories from fat). After 120 days on a HF diet, those mice which developed a well-pronounced obesity (with BWs higher than 50 g) were selected. Thereafter, mice were injected bi-laterally into SGs with either a PYY-, or GFP-encoding rAAV. After the injection, all treated mice were fed a HF diet for 8 weeks. There was a significant difference in weekly FI between rAAV-PYY and rAAV-GFP groups (Fig. 8D). At the end of the 8-week study, the rAAV-PYY-treated mice had lost 24% of their BW compared to the rAAV-GFP control group (Fig. 8E). Reduction in BW was consistent with the weight of visceral white adipose tissue harvested at the end of the overall experiment (Fig. 8F).

## Discussion

Gut hormones play an essential role in maintaining the brain-gut axis by inducing hunger or satiation in a short-term mode. Recently, the expression of several of these peptides was detected in taste cells where they have been shown to modulate taste perception. Little, however, is known about whether these or other gut peptides could accumulate in saliva and whether they could play a functional role mediating satiation. In this study, we provide evidence that the spectrum of metabolically relevant peptides present in murine and human saliva includes gut hormone PYY_3-36_ and that this peptide can be utilized to induced satiation.

Herein, we have shown that salivary PYY_3-36_ enters the oral cavity at least in part from the bloodstream. It is not known whether PYY_3-36_ is selectively transported from blood capillaries, or is non-specifically leaking into the gingival crevicular fluid. What is clear, however, is that salivary and plasma peptide concentrations in humans increase postprandially. In addition, because PYY is also synthesized in the TCs of the CV, it is conceivable that PYY_3-36_ is secreted from these cells into saliva. Using MS analysis, we were unable to detect PYY_1-36_ in human saliva, a result that is readily explained by the action of salivary DPP-IV secreted from salivary glands [30], [31]. On the other hand, no expression of DPP-IV had been detected in TCs inside the taste buds [4] which opens a possibility that there are two distinct pools of PYY: 1) PYY_1-36_ synthesized and contained within the taste buds; 2) salivary PYY_3-36_ derived from plasma. Two PYY moieties could play separate functions: for example, PYY_1-36_ in TCs modulating taste perception by interacting with Y1R expressed in some taste cells [32], while PYY_3-36_ in saliva modulating, in part, feeding behavior by interacting with Y2R in the tongue epithelial cells.

Conceptually, the latter attribute of salivary PYY_3-36_ appears to be redundant considering that PYY enters saliva from plasma, while at the same time inducing satiation through hypothalamic Y2 receptors in a well-described fashion. In the current report, however, we have provided ample experimental evidence showing that the augmentation of salivary PYY_3-36_ indeed reduced FI. The evidence was obtained in feeding behavioral studies using 1) escalating doses of PYY_3-36_, 2) GLP-1 receptor agonist (Exendin-4), 3) selective Y2R antagonist (BIIE0246), 4) other endocrine peptides (GLP-1, amylin), 5) re-feeding-after-fasting, and 6) *ad libitum* feeding paradigms, while utilizing both C57Bl/6J and PYY KO mice models. Moreover, we have shown that one time treatment in mice at the beginning of a dark cycle conducted over twenty consecutive days was sufficient to reduce the rate of BW accumulation. This surprising persistence of the anorexigenic effect can be explained, in part, by a nocturnal feeding pattern in rodents, which is in contrast to the human diurnal feeding. Rodents consume most of the food at the beginning of the dark cycle [17] coinciding with initial effect of PYY oral spray treatment.

To address the issue of stability of the orally applied PYY_3-36_ we developed a completely different treatment paradigm, using rAAV-mediated gene transfer. SG cells exhibit at least two distinct secretory pathways: a predominant regulated one leading to exocrine protein secretion into saliva via zymogen granules and a constitutive one leading to the bloodstream [33], [34]. To benefit from this difference in secretion mechanisms, the transgene to be used was designed to incorporate pre-pro-PYY signal sequences in order for the transgene-encoded PYY peptide to enter the regulated exocrine pathway. Thus, via this secretion pathway, in-between meals the basal level of salivary PYY_3-36_ in treated animals would not significantly exceed the physiological concentrations, hence not affecting appetite. However, upon meal onset, the accompanying increased salivation would induce secretion of PYY_3-36_ stored in SG exocrine granules while activating PYY signaling and inducing faster satiation.

A sustained expression of pre-pro-PYY transgene from the rAAV-transduced cells in the SG resulted in two-fold increase of PYY_3-36_ in saliva with no apparent endogenous secretion into the bloodstream. Surprisingly, this modest increase over physiological levels resulted in significant reduction in 24 hr FI in DIO mice fed a HF diet *ad libitum*. Over the course of 8 weeks vector treated mice lost 24% their BW. In both acute and chronic experiment (Fig. 6G and 8A), we were unable to detect any evidence of salivary PYY diffused retrogradely to plasma in opposite direction to its transport from plasma to saliva. Moreover, considering the salivary milieu enriched with proteases, it is highly unlikely that PYY_3-36_ would stay intact for a significant period of time, sufficient for it to diffuse into plasma.

These data, together with Y2R selective agonist data, point to the oral mucosal epithelial Y2R-positive cells as potential targets for anorexigenic actions of the salivary PYY_3-36_ and suggests the existence of a putative neuronal circuit initiated in the oral cavity. We observed a juxtaposition of Y2R-positive cells and neuronal filaments in the basal cell layer of the murine tongue. Whether this finding observed by IHC methods is reflective of their functional synaptic connection remains to be investigated using more precise electron microscopy methods. However, if such a signaling pathway exists, it might not be inducing an aversive response that follows the peripheral administration of PYY_3-36_ and activation of neurons in the circumventricular organs of the area postrema and intermediate portion of nucleus of a solitary tract [35]. Instead, it would activate somatosensory neurons innervating the receptor field of the tongue epithelia converging with afferent gustatory neuronal pathways. Experiments are in progress to characterize this putative neural network engaged by salivary PYY_3-36_.

Whether the described putative circuit plays an auxiliary role to systemic PYY signaling is a subject of an ongoing investigation. The more parsimonious explanation, however, is that PYY_3-36_/Y2R/G_i_ signaling in K5+ cells plays proliferative role inducing mitotic activity of the progenitor cells in response to the mastication-related loss of keratinized lingual epithelial cells. On the other hand, the G_i_ signaling in K5-progenitor cells could mediate their motility, polarity and migration towards upper layer of keratinized filiform papillae (Fig. 5) [36]. Regardless of the innate physiological functions, it is clear that the augmentation of salivary PYY_3-36_ can be utilized to induce satiation in a simple, pharmacologically relevant way.

In summary, we have shown that the gut satiation peptide PYY_3-36_ is present in saliva where it can play a physiological role in food intake. The anorexigenic effect is apparently mediated through the activation of the specific Y2 receptor expressed in the lingual epithelial cells. We have exploited this putative metabolic circuit to control FI, in a simple and efficient way, suggesting a potential novel therapeutic application for the treatment of obesity.

## Methods

### Mice

These studies (Approval ID #02123, *“Gene Therapy for Obesity”*, and Approval ID #03059, *“Modulation of taste sensitivity by PYY Signaling”*) were approved by the respective Institutional Animal Care and Use Committees (IACUC) at the University of Florida and the NIDCR (NIH). All procedures were done in accordance with the principles of the National Research Council's Guide for the Care and Use of Laboratory Animals. Studies were conducted in male mice housed at 22-24°C in a 12 hours light/dark cycle. Mice had free access to water and food unless indicated otherwise. NPY KO male mice [19] were purchased from Jackson Labs (129-NPY^tm1Rpa^/J), and the PYY KO colony at the UF was derived from the respective breeders [18]. A colony of Y2R KO mice [22] is maintained at the Garvan Institute of Medical Research.

### Mouse saliva collection

Salivation was stimulated as described earlier [37]. Whole saliva was collected for 5 min from the oral cavity into Eppendorf tubes containing 5000 U Kallikrein inhibitor (Biomedicals) and 50 mM DPP-IV inhibitor (Linco Research). Saliva samples were frozen at −80°C until analyzed.

### Human studies

The study (Approval ID #82, *“Characterizing gut hormones in saliva in men and correlating them to amounts in plasma”*) had been approved by the Institutional Review Board (IRB) of the University of Florida. IRB-approved informed written consents were obtained from 5 lean (BMI 19-25) males aged 18–30 with no known diseases. Participants fasted overnight. The next morning, participants' whole saliva samples were collected during fasting and 30 min after eating 450 kcal meals. In each case, saliva was collected using passive drooling approach. The collection tube contained inhibitors as specified for mouse samples; the samples were centrifuged at 4°C to remove debris and frozen at −80°C until analyzed.

### Plasma collection

Blood was collected from facial vein puncture into EDTA-coated tubes (Capiject) containing protease inhibitors as described for saliva collection. Plasma samples were frozen at −80°C until analyzed.

### Plasma and saliva hormone levels

PYY_3-36_ from saliva and plasma was measured by PYY_3-36_ RIA kit (Phoenix Pharmaceuticals). The identification of PYY_3-36_ from human saliva was performed by reverse-phase high performance liquid chromatography (RP-HPLC) followed by Matrix-Assisted Laser Desorption Ionization – Time of Flight (MALDI-TOF) mass spectrometry (MS). PYY_3-36_ synthetic peptide (Bachem) was used for calibration of all purification steps. Briefly, saliva was pre-purified using a Sep-Pak C18 cartridge (Waters) followed by purification on RP-HPLC uBondapak C18 column (Waters) as described [38]. The PYY_3-36_-containing fraction was collected, air dried and reconstituted in 0.1% formic acid in 50% acetonitrile/water prior to analysis with MALDI-TOF as described [39], [40].

### Immunoflurescent localization

For specific information on the source of all antibodies, dilutions, and controls please see Table 1.

**Table 1:** Antibodies used for immunolocalization studies.

Antibody	Host	Supplier	Dilution	Specificity/Control
Anti-PYY	Rabbit	Abcam (Cambridge, MA, USA; cat. No. ab22663)	1∶2000	Staining absent when primary or secondary antibodies omitted. Staining visible when PYY KO tissues were used due to partial cross reactivity with NPY (25%). Staining visualized in NPY KO tissues. Use of this antibody has been reported previously.
Anti-Y2R	Rabbit	Neuromics (Edina, MN, USA; cat. No. RA14112)	1∶3000 (using TSA Kit)	Staining absent when primary or secondary antibodies omitted, or in NPY Y2 receptor KO. Use of this antibody has been reported previously. Western blot analysis on hippocampal membrane fractions revealed a single band of 44 kDa (Stanic et al, J Comp Neurol 2011, v.519, p. 1219-1257).
Anti-NCAM	Rabbit	Millipore (Temecula, CA, USA; cat. No. AB5032)	1∶500	Staining absent when primary or secondary antibodies omitted.
Anti-Keratine 5	Rabbit	Covance (Emerit, CA, USA; cat. No. PRB-160P)	1∶1000	Staining absent when primary or secondary antibodies omitted.

#### PYY

Tissues were harvested from overnight fasted animals, immersed in Bouin's fixative for 8 hrs at 4°C, dehydrated, paraffin-embedded and sectioned in a rotary microtome at 4 mM thicknesses. For PYY immunolocalization, tissues were blocked with 3% H_2_O_2_ in methanol followed by antigen retrieval with Trypsin (DIGEST-ALL 2, Invitrogen), blocking with 5% natural donkey serum in TNT (0.1 M Tris-HCl, pH 7.5, 0.15 M NaCl and 0.05% Tween 20), overnight incubation with rabbit anti-PYY in TNT, blocking with Image-iT® FX Signal Enhancer (Invitrogen) and detection with donkey anti-rabbit Alexa Fluor 488 in TNT (1∶1000, Invitrogen).

#### Y2R

Tissues were harvested from fasted animals and immediately frozen. 4 mM thick sections were cut using a cryostat (Leica CM3050 S; Leica Microsystems, Nussloch GmbH, Germany) and then fixed in 4% paraformaldehyde for 10 min. Y2R immunolocalization was done with the TSA kit (Perkin Elmer). Tissues were blocked in 0.9% H_2_O_2_ in TBS for 30 min followed by blocking with TNB (0.1 M Tris-HCl, pH 7.5, 0.15 M NaCl and 0.5% Blocking Reagent from Perkin Elmer), incubation with rabbit anti-Y2R in TBS, incubation with goat anti-rabbit MACH 2 HRP-polymer (Biocare Medical) and detection with Fluorescein provided in the TSA kit (1∶300).

#### Cytokeratin-5, NCAM

The same as for Y2R detection protocol was followed, but after incubation with primary antibodies, sections were blocked with Image-iT® FX Signal Enhancer (Invitrogen) and incubated with goat anti-rabbit Cy3 (1∶800, Jackson Immunoresearch) for visualization. All sections were counterstained with DAPI.

### Relative quantitative RT-PCR analysis

RNA extraction, purification, cDNA synthesis and RT-PCR amplification was done as described in Aslanadi et al [41]. Samples from each animal were treated individually through the end of the analysis.

### PYY_3-36_ acute augmentation studies

The concentration of PYY_3-36_ in the oral cavity was acutely increased by utilizing an oral spray. One ml total volume sterile vials were obtained from Sephora. Murine PYY_3-36_ (Bachem) was diluted in sterile H_2_O. All 8–10 weeks old mice were individually housed. Mice were conditioned to the oral spray with vehicle after 24 hours fast on three separate occasions. Groups were randomized by FI and BW. Prior to the study day, mice were fasted for 24 hours, the oral spray in a form of a single puff containing PYY_3-36_ (concentration ranging from 3 to 18 mg/100 g BW, as specified), GLP-1 (3 mg/100 g BW), Exendin-4 (10 mg/100 g BW), or vehicle was applied without any sedation. The applied dose was estimated using calculated average volume (∼30 ml) delivered per puff. Food was provided 10 min after the spray was applied and the amount consumed was measured at either 1 hr after the treatment, or at several time points (1, 2, 4, 6, 12, 18, and 24 hours). Taking into account the nocturnal feeding pattern, the experiment was conducted during the night with the first measurement taken at 2000 hrs. Each experiment was done at least 3 times in a crossover manner with 8 mice per group. For the Y2R antagonist study, BIIE0246 compound (Tocris Bioscience, Ellisville, MO) was dissolved in 100% EtOH (0.2 mM) and the stock solution was mixed with PYY_3-36_ aqueous solution at the 50∶1 molar ratio (BIIE0246/PYY_3-36_) with the final concentration of 2% EtOH in the mixture. Prior to this experiment, mice were conditioned to 2% EtOH spray on three separate occasions. Wherever practical, the behavioral experiments were conducted in a blind fashion with the personnel uninformed about the treatment regimen.

### *In vitro* studies

Genetically engineered human intestinal NCI-H716 cells were utilized to test PYY transgene as described previously by Tang and Sambanis [29]. Briefly, cells were subjected to a differentiation protocol and then transduced with rAAV-PYY, or rAAV-GFP (multiplicity of infection, M.O.I. of 10^4^). Forty eight hours later, cells were washed and starved overnight in a minimal media (DMEM, GIBCO) containing 5 mM glucose and 1% fetal bovine serum. Cells were then washed twice with minimal media for 2 hrs to stabilize their basal secretion followed by stimulation with 2% meat hydrolysate (Sigma) in minimal media for one hour. For the following 4 hrs, cells were incubated in minimal media, with consecutive replacements of the media every hour. Harvested media were assayed for PYY using the above-described EIA kit.

### Gene transfer experiments

Long-term chronic expression of PYY_3-36_ was achieved by rAAV-mediated PYY gene transfer targeted to SG. rAAV was constructed encoding murine pre-pro-PYY cDNA driven by a strong constitutive CMV/b-actin promoter (Fig. 7A). PYY-, and GFP-expressing cassettes were pseudotyped into rAAV5 capsids as having higher transduction efficiencies in murine SG [42]. The viral vector production, purification and titering were done as described earlier [43]. A single dose of 100 ml containing 1×10^10^ vector genomes was administered bilaterally into each duct of the SG, as described previously [42].

### Statistical analysis

Fixed effects ANOVA was used to determine overall model adequacy of mouse weight as the response variable and treatment type as the single factor in the experiment. Pairwise comparisons of the factor were carried out using a Fisher's Protected LSD with a type I error rate of P≤0.05. Alternatively, statistical analysis was conducted using un-paired Student's t-test with significance at P<0.05.
